# Re-appraisal of *Nertera* (Rubiaceae) in Taiwan

**DOI:** 10.3897/phytokeys.182.70685

**Published:** 2021-09-20

**Authors:** Wei-Chih Chen, Chih-Chiang Wang, Kun-Cheng Chang

**Affiliations:** 1 Graduate Institute of Bioresources, National Pingtung University of Science and Technology, Pingtung 912, Taiwan; 2 Department of Forestry, National Pingtung University of Science and Technology, Pingtung 912, Taiwan; 3 Department of Forestry and Nature Resources, National Chiayi University, 300 Syuefu Rd., Chiayi city 600, Taiwan

**Keywords:** *
Nertera depressa
*, *
Nertera granadensis
*, *
Nertera nigricarpa
*, *
Nertera taiwaniana
*, Rubiaceae

## Abstract

A revision of *Nertera* (Rubiaceae) in Taiwan was carried out by classical taxonomic methods and the presence of two endemic species was confirmed. Only one species, misapplied as *N.granadensis*, had been reported in the second edition of “Flora of Taiwan”, but there were two additional endemic species in this genus: *N.nigricarpa* and *N.taiwaniana* confirmed. *Nerteranigricarpa* is characterised by the entire leaf, purple-black petals, black fruits and dark-purple seeds with raised striate. *Nerterataiwaniana* has leaves with undulated margins, yellowish-green petals, red fruits and yellow-white seeds without striate. *N.granadensis* is excluded from the flora of this Island.

## Introduction

There are six known species in the genus *Nertera* Banks ex Gaertn. in the family Rubiaceae in Australia, New Zealand, South America, Indonesia (Java), China, Taiwan and the Philippines (Chen and Taylor 2011). Moreover, Thompson (2010) suggested about 15 species in the genus. Hayata (1908) first described *N.nigricarpa* Hayata and reported that it is endemic to Taiwan and distributed at mid- to high-altitudes across the Island. Later, Hayata (1918) provided line drawings of *N.nigricarpa*. Masamune (1938) first described *N.taiwaniana* Masam., which has red fruits and was only known from the Jingshueiying area in southern Taiwan. However, *N.taiwaniana* has recently been discovered near Yuanyang Lake in northern Taiwan and at Lijia Industry Road in eastern Taiwan. Yamamoto (1938, 1940) believed that *N.depressa* Banks & Sol. ex Gaertn. in the Philippines and *N.taiwaniana* in Taiwan were the same species. Chao (1978) included both *N.depressa* and *N.nigricarpa* in the first edition of “Flora of Taiwan” and also treated *N.taiwaniana* as being a synonym of *N.depressa* in accordance with Yamamoto (1938, 1940), which was followed by Ko (1999). Liu and Yang (1998) only recorded one species, *N.granadensis* (Mutis ex L. f.) Druce, in the second edition of “Flora of Taiwan” and treated *N.depressa* and *N.nigricarpa* as synonymous.

## Materials and methods

This study is based on field observations and detailed examinations of herbarium specimens. Specimens examined included those from the Herbaria CHIA, HAST, TAI, TAIF and TCF. The other type specimens were accessed as digital images of *Nerteradepressa* and *N.granadensis* from Herbaria LINN and WELT. Morphological comparisons of fresh leaves, flowers, fruits and seeds were observed and stereomicroscopic pictures were taken of plants from Taiwan. Their sizes were measured by a digital caliper.

## Results and discussion

**Figure 1.**
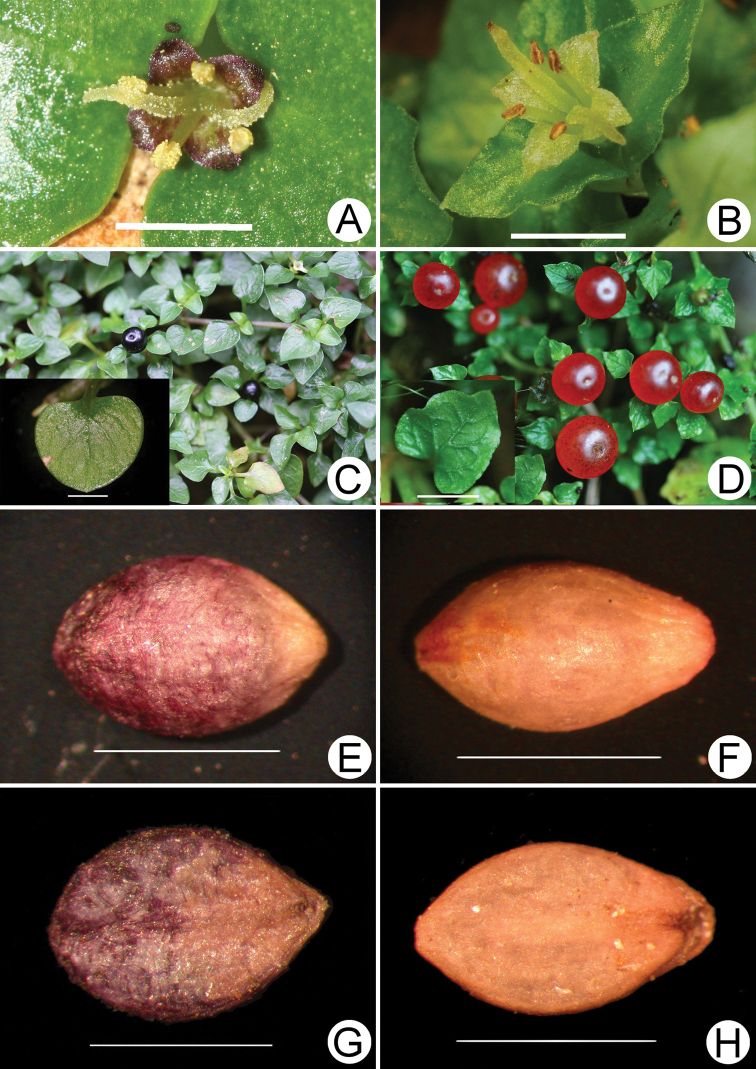
Photographs of *Nerteranigricarpa* Hayata and *N.taiwaniana* Masam **A** flower of *N.nigricarpa*. **B** flower of *N.taiwaniana***C** fruits and leaf shape of *N.nigricarpa***D** fruits and leaf shape of *N.taiwaniana***E** top side of *N.nigricarpa* seed **F** top side of *N.taiwaniana* seed **G** underside of *N.nigricarpa* seed **H** underside of *N.taiwaniana* seed. Scale bars: 2 mm.

Based on field observations and detailed examinations, we verified that the black-fruited *Nertera* and the red-fruited *Nertera*, native to Taiwan, are different species (Fig. 1). *Nerterataiwaniana* has leaves with undulating margins and secondary veins that are distinctly convex on the upper surface, yellowish-green petals, red fruits and yellow-white seeds without striate surfaces. *Nerteranigricarpa* is characterised by leaves entire without undulating margins and secondary veins which are not apparent on the upper surface or, if apparent, then only slightly convex, purple-black petals, black fruits and dark-purple seeds with raised striate.

Furthermore, after studying the lectotypes of *Nerteragranadensis* (*Mutis s.n.*, LINN) and a live individual, growing in its country of origin, Columbia, we found that the leaves of *N.granadensis* are nearly fleshy, with no apparent veins on both surfaces, without undulating margins, with an obtuse to acute apex and obtuse to shallowly truncate base and with red fruits that have no black spots. Therefore, *N.granadensis* is morphologically distinguishable from *N.nigricarpa* and *N.taiwaniana*. Although, the fruits of both *N.granadensis* and *N.taiwaniana* are red, *N.taiwaniana* possesses leaves that are membranous to papery with distinctly convex veins, an acute apex, a cordate or truncate base and fruits with black spots.

*Nerteragranadensis* has often been referred to as *N.depressa* Banks & Sol. ex Gaertn, the type of the genus (Chen and Taylor 2011). Chao (1978) treated *Nerterataiwaniana* as a synonym of *N.depressa*. We also compared at syntype of *N.depressa* (Banks & Solander *s.n*., WELT SP063852). *Nerteradepressa* possesses slightly fleshy leaves with no apparent veins, no undulated margins, an acute apex, an obtuse or shallowly cordate base and fruits that are red without black spots. In particular, the top hollow of *N.depressa*’s fruit is black. This characteristic is never found on the species native to Taiwan and *N.granadensis*.

Considering the current evidence, the endemic species, *N.nigricarpa* and *N.taiwaniana* were proposed herein readily distinguished from *N.granadensis* and *N.depressa*.

### Key to *Nertera* species of Taiwan and the excluded species *Nerteragranadensis*.

**Table:** 

1	Fruits black; corolla purple-black; leaf margins entire without undulating, veins not apparent or slightly convex on upper leaf surface	***Nerteranigricarpa* Hayata**
–	Fruits red; corolla yellowish or yellowish-green; leaf margins undulating.	
2	Mature fruits red with black spots; corolla yellowish-green; leaves membranous to papery, veins distinctly convex on upper surface	***Nerterataiwaniana* Masam**
–	Mature fruits red without black spots; corolla yellowish; leaves nearly fleshy, veins usually not apparent or only slightly convex on upper surface	***Nerteragranadensis* Druce**

### Taxonomic treatment

#### Nertera

Taxon classificationPlantaeGentianalesRubiaceae

Banks ex Gaertner, Fruct. Sem. Pl. 1: 124. 1788 nom. cons.

CA986DF7-E4EF-5A9C-8CB3-0EA28B5EA32E

Erythrodanum Thouars, Mélang. Bot. 9: 41. 1811.
Gomozia Mutis ex L. f., Suppl. Pl. 17, 129. 1781.

##### Note.

About 7–15 species in tropical Asia, Pacific Islands and America; 2 species in Taiwan.

#### Nertera nigricarpa

Taxon classificationPlantaeGentianalesRubiaceae

Hayata, Journ. Coll. Sci. Univ. Tokyo 25 (19): 115. 1908 (Fl. Mont. Form.); Icon. Pl. Form. 7: 32. 1918; Chao, Fl. Taiwan 4: 315. pl. 1020. 1978; Ko, Fl. Reipubl. Popularis Sin. 71 (2): 164.

9DCE0E7A-6EE0-54DF-9EC2-9B59ED79B911

**Figure 2.**
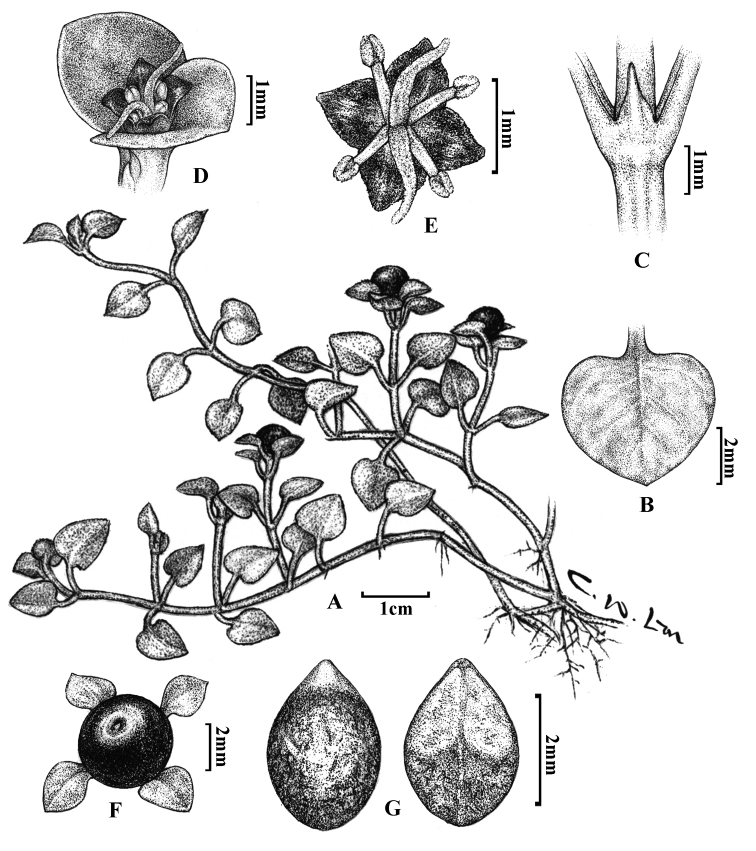
*Nerteranigricarpa* Hayata **A** habit **B** leaf **C** stipule **D** inflorescence **E** flower **F** fruit and **G** seeds.

Figs 1a, c, e, g , 2 ; Table 1

Nertera granadensis auct. non Druce: Liu & Yang, Fl. Taiwan Second 4: 306. pro parte.

##### Notes.

Creeping glabrous herbs; branches slender, 5–20 cm long. Leaves reniform to broad-ovate or deltoid, papery to thick-papery, 6–13 mm long, 4–8 mm wide; apex obtuse to acute, base cordate or truncate, margins entire without undulating; petioles 2–7 mm long; secondary veins 2–3 on each side of mid-vein, usually not apparent on upper surface but, if visible, slightly convex, slightly impressed on lower surface; stipules lanceolate to triangular, membranous, ca. 1.3 mm long and 0.9 mm wide. Flowers sessile, solitary, terminal, ca. 1.5 mm long and 1.5 mm wide; calyx truncate, glabrous; corolla purple-black, lobes 4, deltoid to ovate, ca. 0.6 mm long and 0.5 mm wide, apex acute; stamens 4, anthers ovate, ca. 0.36 mm long and 0.2 mm in diameter, filaments ca. 0.4 mm long; ovary ellipsoid, two-celled, each with one ovule; two styles, free, ca. 1 mm long. Fruit globose drupe, 3–5 mm in diameter, black at maturity; two seeds, dark-purple, ovate to long-ovate, 2–3 mm long, 1.7–2 mm wide, surfaces striated.

**Table 1.** Comparison of Taiwanese *Nertera* native species and the excluded species *Nerteragranadensis*.

	* Nertera granadensis *	* Nertera taiwaniana *	* Nertera nigricarpa *
**Leaf** Texture	Nearly fleshy	Membranous to papery	Papery to thick-papery
Veins	Usually not apparent or, if apparent, slightly convex on upper surface	Distinctly convex on upper surface	Usually not apparent or, if apparent, slightly convex on upper surface
Margins	Undulate	Undulate	Entire without undulating
**Flower** Colour	Yellowish	Yellowish-green	Purple-black
**Fruit** Colour	Red without black spots	Red with black spots	Black
**Seed** Colour	Yellow-white	Yellow-white	Dark-purple
Ornamentation	Not striate	Not striate	Striate

##### Distribution and habitat.

*Nerteranigricarpa* is endemic in Taiwan, at medium altitudes throughout the Island.

##### Specimens examined.

**Taiwan**, **Taipei**: Bunzangun, 6 Aug 1938, *T. Nakamura 673* (TAI); Mt. Chiamu, 11 May 1935, *N. Fukuyama 19242* (TAI); Hsintien, 15 Dec 2000, *Chen* et al. *3771* (TAIF); **Hsinchu**: Mt. Tapachienshan, 6 Sept 1993, *C. L. Huang 71* (HAST), 1 Nov. 1996, *C. M. Wang 2370* (TAIF); **Taichung**: Suyuan, 13 Jan 2000, *Y. P. Cheng 2950* (TAIF); Mt. Pahsien, 7 Aug 1959, *T. I. Chung 2697* (HAST); Mt. Amma, 20 Oct 1957, *T. S. Liu 197* (HAST); **Nantou**: Mayfeng to Sungkang, 21 Jan 1986, *C. I Peng 9079* (HAST); Yuanfeng, 5 Jul 2000, *Y. P. Cheng 3253* (TAIF); Tunyuan to Yunhai, 20 May 1993, *C. C. Liao 1299* (HAST); Tatachiaanpu to Lulinshanchuang, 7 Aug 1991, *W. P. Leu 1208* (HAST); Kuankao to Patungkuan, 4 Jul 1985, *C. I Peng 8181* (HAST); **Chiayi**: Shimeng Valley, 6 Feb 2007, *K. C. Chang* et al. *3802* (TCF); Mt. Ali, 17 Dec 1939, *Nakamura & Yamamoto 4133* (TAI); 10 Oct 1983, *C. I Peng 6018* (HAST); **Kaohsiung**: Chungtzukuan, 19 Dec 2000, *S. J. Yang 29999* (TAIF); Chuyunshan, *H. L. Ho 899* (HAST); **Pingtung**: Mt. Peitawu, 23 May 1918, *E. Matuda 1710* (TAI); 7 Feb 1990, *C. I Peng 13276* (HAST); 5 May 2004, *K. C. Chang 2419* (CHIA); **Ilan**: Fanfan, 27 Aug 1918, *E. Matuda 430* (TAI); Tananao, 21 Jul 1929, *S. Suzuki 628* (TAI); Mt. Chialoshan, 27 Sept 1930, *S. Suzuki 6220* (TAI); Mt. Chililo, 5 Aug 1928, *S. Suzuki s. n*. (TAI); Mt. Taiping, 25 Jul 1929, *S. Suzuki 920* (TAI); 26 Aug 1962, *Kao et al. 4961* (TAI); 2 Mar 1966, *Kao & Chuang 4093* (TAI); 26 Aug 1962, *M. T. Kao 4691* (HAST); Chililo-Hsulawa, 16 Jul 1932, *S. Suzuki 7206* (TAI); Kiyanrawa, 25 Sept 1930, *S. Suzuki 6182* (TAI); **Hualien**: Hoping Logging Trail, 15 Apr 2006, *S. W. Chung* 8489 (TAIF); Mt. Chingshui, 9 Sept 1939, *T. Nakamura 3780* (TAI); Tailoku-Tailokutaishan, 15 Jun 1933, *A. T. Hsieh s. n.* (TAI); Mt. Mukua, 7 Aug 1940, *T. Nakamura 4526* (TAI); 23 Sept 1984, *C. I Peng 7269* (HAST); Tayulin, 17 Oct 2004, *J. H. Lii 1135* (TAI); Mt. Lintien, 12 Feb 1962, *J. M. Chao 810* (TAI); **Taitung**: Siangyang, 18 Mar 2006, *K. C. Chang & C. C. Wang 3069* (TCF).

#### Nertera taiwaniana

Taxon classificationPlantaeGentianalesRubiaceae

Masam., Trans. Nat. Hist. Soc. Form. 28: 144. 1938.

67BC7BD2-7A54-5EAA-8445-D957FB69316F

**Figure 3.**
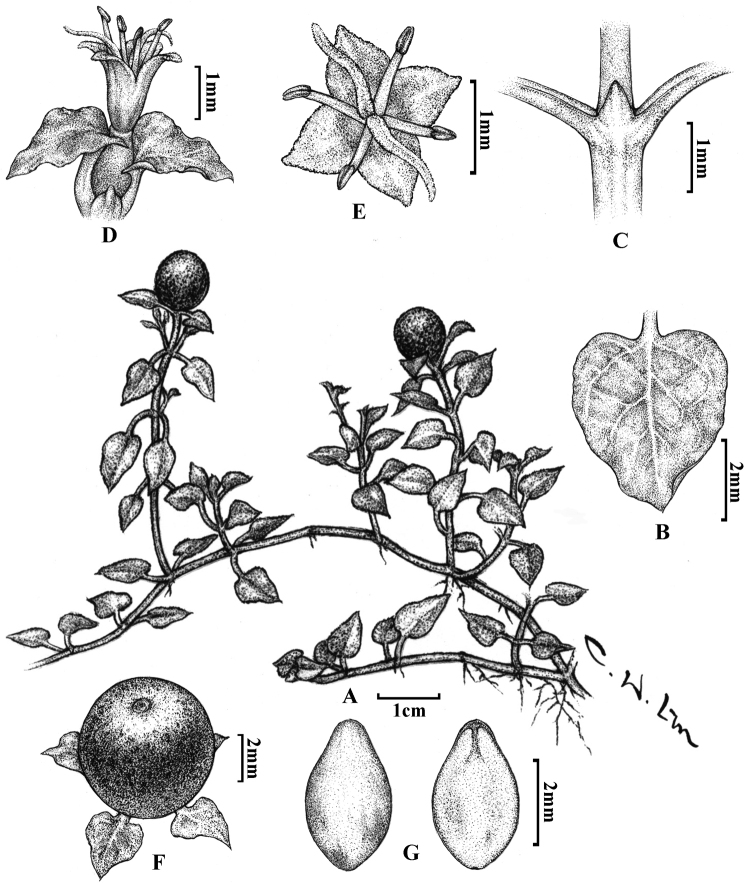
*Nerterataiwaniana* Masam **A** habit **B** leaf **C** stipule **D** inflorescence **E** flower **F** fruit and **G** seeds.

Figs 1b, d, f, h , 3 ; Table 1

Nertera granadensis auct. non Druce; Liu & Yang, Fl. Taiwan 2^nd^ 4: 306. pro parte; Chen & Taylor, Fl. China 19: 257. 2011. syn. N.taiwaniana.
Nertera depressa auct. non Banks & Sol. ex Gaertn: Yamamoto, Journ. Soc. Trop. Agr. 10: 276. 1938, 12: 24. 1940; Chao, Fl. Taiwan 4: 315. pro parte; Ko, Fl. Reipubl. Popularis Sin. 71 (2): 164. syn. N.taiwaniana.

##### Notes.

Creeping herbs; branches slender, 5–15 cm long, glabrous. Leaves opposite, deltoid to ovate, membranous to papery, 4–12 mm long, 2–8 mm wide, apex acute to acuminate, base cordate or truncate, margins entire, more or less undulate; petioles 1.5–6 mm long; secondary veins 2–3 on each side of mid-vein, distinctly convex on upper surface, not apparent on lower surface; stipules triangular, membranous, ca. 1.5 mm long and 1.5 mm wide. Flowers sessile, solitary, terminal, ca. 2 mm long and 1.8 mm wide; calyx truncate, glabrous; corolla yellowish-green, lobes 4, deltoid to ovate, ca. 0.7 mm long and 0.6 mm wide, apex acute; stamens 4, anthers oblong, ca. 0.29 mm long and 0.15 mm in diameter, filaments ca. 0.5 mm long; ovary ellipsoid, two-celled, each with one ovule; two styles, free, ca. 1 mm long. Fruit globose drupe, 4.5–6.5 mm in diameter, red with black spots at maturity; seeds 2, yellowish-white, long-ovate to spathulate, 2.5–3.5 mm long, 1.5–2 mm wide, surfaces smooth.

##### Distribution and habitat.

*Nerterataiwaniana* is endemic in Taiwan. Growing on hillsides at medium altitudes in the eastern and southern parts of the Island.

##### Specimens examined.

**Taiwan, Pingtung**: Chunjih Hsiang, 30 Dec 1999, *C. I Peng 17902* (HAST); Tahan Forest Road, 26 Jul 2001, *Y. Y. Huang 554* (HAST); Chinshuiying, 23 Jun 1999, *K. F. Chung 1348* (HAST); 18 May 2008, *K. C. Chang & C. C. Wang 4447* (TCF); 6 Jun 2009, *K. C. Chang & C. C. Wang s. n.* (TCF); **Ilan**: Shenmihu, 28 Dec 1987, *Y. M. Hsu 554* (TAI); Chialohu, 10 May 2002, *C. I Huang 815* (HAST); Mt. Taiping, 22 Dec. 1995, *C. H. Chen 1519* (HAST); Mt. Fanpao, 17 Apr 1996, *C. C. Liao 1798* (HAST); **Taitung**: Mt. Sung, 30 Aug 1932, *S. Suzuki s. n.* (TAI); 13 May 1988, *S. Y. Lu 22900* (TAIF); Mt. Kutzulun, 20 Jul 1937, *H. Simizu 3888* (*Nerterataiwaniana*, holotype: TAI!).

###### Excluded species to the Flora of Taiwan

#### Nertera granadensis

Taxon classificationPlantaeGentianalesRubiaceae

(Mutis ex L. f.) Druce, Rep. Bot. Soc. Exch. Club Brit. Isles 1916: 637. 1917; Chen & Taylor, Fl. China 19: 257. 2011. excl. Taiwan – Gomozia granadensis Mutis ex L. f., Suppl. Pl. 129. 1781.

14AB6D70-C3BA-508E-8E4C-E761DDC7E6F5

##### Specimens examined.

**Colombia. South America**: no date, *Mutis s.n.* (LINN).

##### Distribution and notes.

*Nerteragranadensis* has an unusually extensive transcontinental distribution surrounding the Pacific Ocean, occurring from New Zealand subantarctic Islands (Tristan da Cunha), South America (Argentina, Bolivia, Chile, Colombia, Ecuador, Peru, Venezuela), Central America (Costa Rica, El Salvador, Guatemala, Honduras, Nicaragua, Panama), North America (Mexico) and in Hawaii, eastern Australia, Indonesia, Malaysia, Papua New Guinea and the Philippines. As we observed that the morphology of *Nertera* populations differed between regions, further and more detailed research is necessary for classification. Considering the current evidence, we suggest that *N.granadensis* is excluded from the Flora of Taiwan.

## Supplementary Material

XML Treatment for Nertera

XML Treatment for Nertera nigricarpa

XML Treatment for Nertera taiwaniana

XML Treatment for Nertera granadensis
